# Counting *Caenorhabditis elegans*: Protocol Optimization and Applications for Population Growth and Toxicity Studies in Liquid Medium

**DOI:** 10.1038/s41598-018-19187-3

**Published:** 2018-01-17

**Authors:** Leona D. Scanlan, Steven P. Lund, Sanem Hosbas Coskun, Shannon K. Hanna, Monique E. Johnson, Christopher M. Sims, Karina Brignoni, Patricia Lapasset, Elijah J. Petersen, John T. Elliott, Bryant C. Nelson

**Affiliations:** 1 National Institute of Standards and Technology, Material Measurement Laboratory – Biomolecular Measurements Division, 100 Bureau Drive, Gaithersburg, MD 20899 United States; 2 National Institute of Standards and Technology, Information Technology Laboratory – Statistical Engineering Division, 100 Bureau Drive, Gaithersburg, MD 20899 United States; 3 National Institute of Standards and Technology, Material Measurement Laboratory – Biosystems and Biomaterials Division, 100 Bureau Drive, Gaithersburg, MD 20899 United States; 4 National Institute of Standards and Technology, Material Measurement Laboratory – Chemical Sciences Division, 100 Bureau Drive, Gaithersburg, MD 20899 United States; 5 Department of Pharmacognosy, Faculty of Pharmacy, Gazi University, 06330 Ankara, Turkey; 6 California EPA Department of Pesticide Regulation, Sacramento, CA 95814 United States

## Abstract

The nematode *Caenorhabditis elegans* is used extensively in molecular, toxicological and genetics research. However, standardized methods for counting nematodes in liquid culture do not exist despite the wide use of nematodes and need for accurate measurements. Herein, we provide a simple and affordable counting protocol developed to maximize count accuracy and minimize variability in liquid nematode culture. Sources of variability in the counting process were identified and tested in 14 separate experiments. Three variables resulted in significant effects on nematode count: shaking of the culture, priming of pipette tips, and sampling location within a microcentrifuge tube. Between-operator variability did not have a statistically significant effect on counts, even among differently-skilled operators. The protocol was used to assess population growth rates of nematodes in two different but common liquid growth media: axenic modified *Caenorhabditis elegans* Habitation and Reproduction medium (mCeHR) and S-basal complete. In mCeHR, nematode populations doubled daily for 10 d. S-basal complete populations initially doubled every 12 h, but slowed within 7 d. We also detected a statistically significant difference between embryo-to-hatchling incubation period of 5 d in mCeHR compared to 4 d in S-basal complete. The developed counting method for *Caenorhabditis elegans* reduces variability and allows for rigorous and reliable experimentation.

## Introduction

Alternative model organisms such as the nematode *Caenorhabditis elegans* are increasingly used to study the toxicity of chemicals^1–3^. Toxicity ranking using *C. elegans* is as predictive as using rat or mouse models for determining LD_50_ values, and the *C. elegans* model may also be predictive for developmental toxicity in humans^2,4–6^. An evolving movement in the United States, spurred by legislation on cosmetic safety testing in Europe, calls for expanding the use of alternative test models and methods to reduce the use of mammals in chemical safety testing, risk assessment and regulatory decision-making^7–9^. The Interagency Coordinating Committee on the Validation of Alternative Methods in conjunction with the National Toxicology Program Interagency Center for the Evaluation of Alternative Toxicological Methods has been charged with coordinating the development of alternative toxicological testing methods and with establishing the framework for scientific validation of proposed methods^10,11^. In addition, the Frank R. Lautenberg Chemical Safety for the 21st Century Act mandates that the U.S. Environmental Protection Agency explore “non-vertebrate” models for toxicity testing (https://www.epa.gov/assessing-and-managing-chemicals-under-tsca/frank-r-lautenberg-chemical-safety-21st-century-act). One tenable path toward the replacement of animals in testing is to investigate the utility of well-characterized invertebrate models, such as *C. elegans*. It is therefore conceivable that *C. elegans* may be incorporated into tiered chemical testing strategies^1^.

Our laboratory is heavily involved in establishing the use of *C. elegans* as a standard model organism for testing the toxicity of nanomaterials and chemicals of commercial interest^3,12^. However, sensitive measurement tools, robust methods and standardized protocols for reliable molecular and toxicological investigations are lacking when it comes to counting methods for the nematode in liquid culture. In order to expand the use of *C. elegans* in toxicological testing, protocols and experimental methods that detail and enumerate specific aspects of nematode culturing, sampling and toxicant exposure are essential. In many reports, counts of live *C. elegans* in liquid cultures are often roughly approximated or not listed. The number of *C. elegans* in an exposure system can impact biological or toxicological responses; the number of cells in culture, for example, can have a substantial impact on nanotoxicity results and EC_50_ values^13,14^. It has been reported that the number and stage of live nematodes used during experimentation, including culture expansion and toxicant exposure, is a critical factor in toxicological measurements^1^. Procedures describing how nematode counts were determined, or how the correct number of *C. elegans* were transferred from one system to another (*e.g*., from a culture vessel to an exposure vessel) are often not clearly stated. This makes it challenging to verify or reproduce experiments, a current key topic in biological research^15,16^. Representative examples of unreported or inadequately described nematode counting protocols can be found in research from neurotoxicology^17–19^, environmental toxicology^20–23^, calorie-restriction^24–26^ and genotoxicology^27,28^.

There currently exists no recognized reference protocol for counting nematodes in liquid media^29^. Laboratories equipped with automated high-throughput sorting and counting instrumentation, such as the COPAS™ Biosort flow cytometry instrument, are able to rapidly count and enumerate nematode stages in liquid cultures^30^. Limitations with flow techniques include effects of ambient temperature and humidity and introduction of artifacts from improper gating or handling^5,31^. COPAS instrumentation is also very expensive, and is not widely available in many laboratories, which limits its use for toxicity testing with *C. elegans*^32^.

To address these issues, we herein describe the development of a robust protocol for counting live nematodes in two commonly used liquid media: modified *C. elegans* Habitation and Reproduction medium (mCeHR)^33,34^, an axenic liquid medium, and S-basal complete, a common medium that uses *Escherichia coli* as a food source. Potential sources of variability in the nematode counting process were identified and experimentally examined. The resulting data were used to develop a standard protocol for nematode counting in liquid media that allows for increased confidence in the counting process. The standardized protocol was then used in proof-of-concept studies to monitor the proliferation of sibling nematode cultures in the two different liquid growth media over a three-week period. The population growth rate experiments were conducted twice, with minor differences, to assess the repeatability of the findings. The standardized counting protocol provided greater assurance that the observed differences in nematode count between culture types were due to differences in population growth and not in measurement technique. Detailed methods to enable reproduction of the reported results are given, and recommendations on best practices for counting nematodes using optical microscopy are presented.

## Results

### Tests of variability

**Figure 1:**
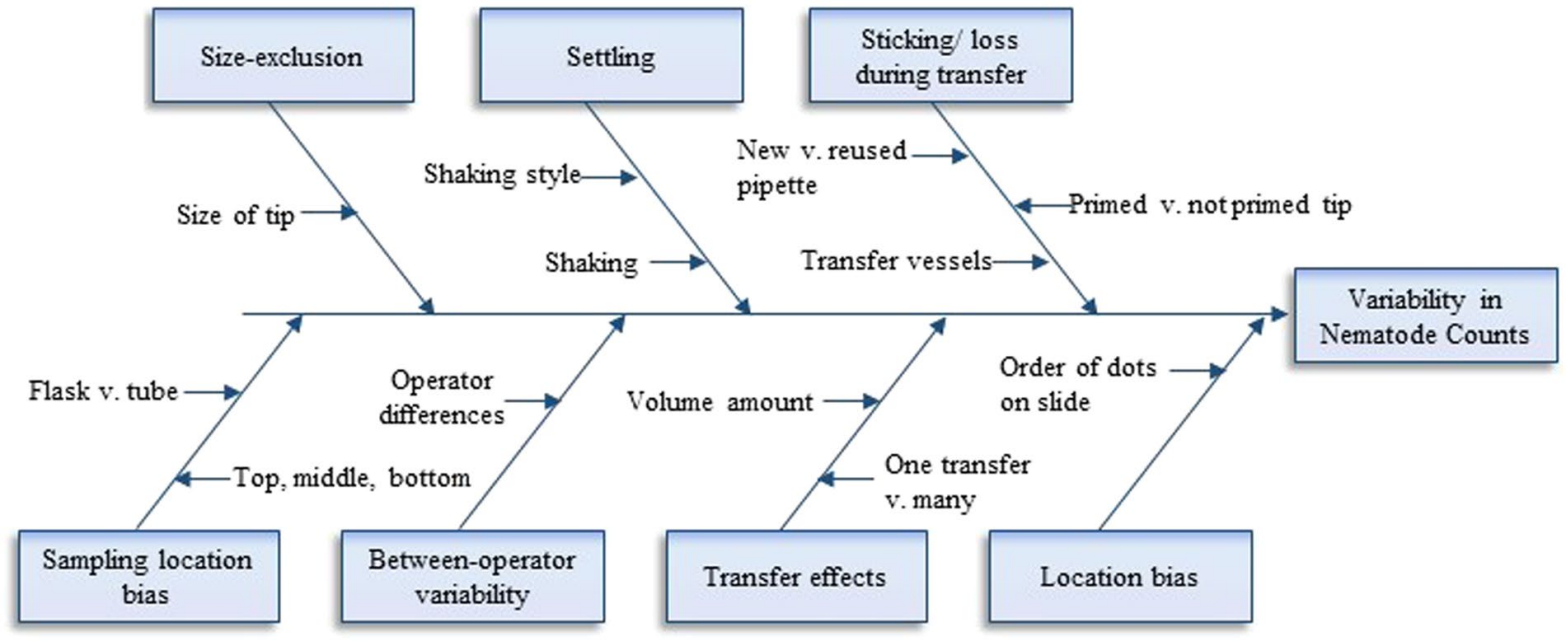
Cause and Effect Diagram of Variability in Nematode Counting. Potential effects on nematode count are described in blue boxes; causes are shown with arrows. Twelve different causes of variability were tested in 14 different tests.

**Figure 2:**
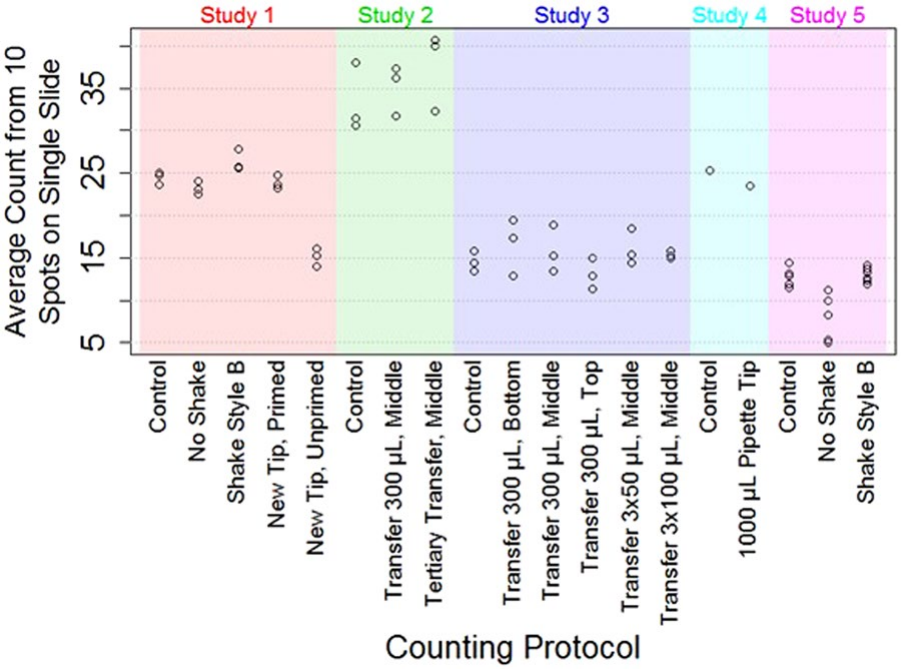
Overview of Data Collected for the Counting Protocol Investigation. The vertical position of each dot depicts the average nematode count across 10 dots distributed on a single slide (with the exception of the 1000 µL pipette tip, for which counts were scaled by 150/500 to account for differences in volume and dilution fraction and for which only 6 replicate measurements were collected). The horizontal position of each dot corresponds to the counting protocol used while counting. The labels along the x-axis indicate the manner in which the tested protocol deviated from the standard counting protocol (called “Control”). The colored background distinguishes separate sample preparations and counting sessions, thus differences due to variations in counting protocol can only be inferred by comparison between results within a single colored region. Experiments were conducted on different days (Study 1, etc). Note: The shaking test was repeated (Study 5) due to concerns about inadvertent shaking during Study 1.

**Figure 3:**
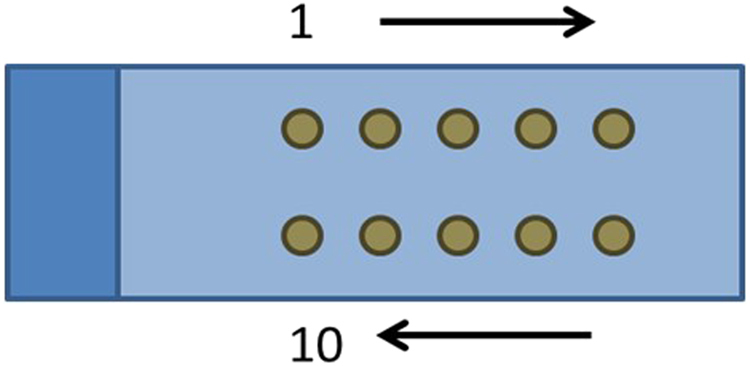
Order of Counting-Replicate (“dot”) Placement on Microscope Slide. Two (2) μL aliquots of nematode culture were placed on a clean microscope slide in the order indicated, from the top left corner to the top right, and then from the bottom right corner to the bottom left, unless indicated otherwise.

**Figure 4:**
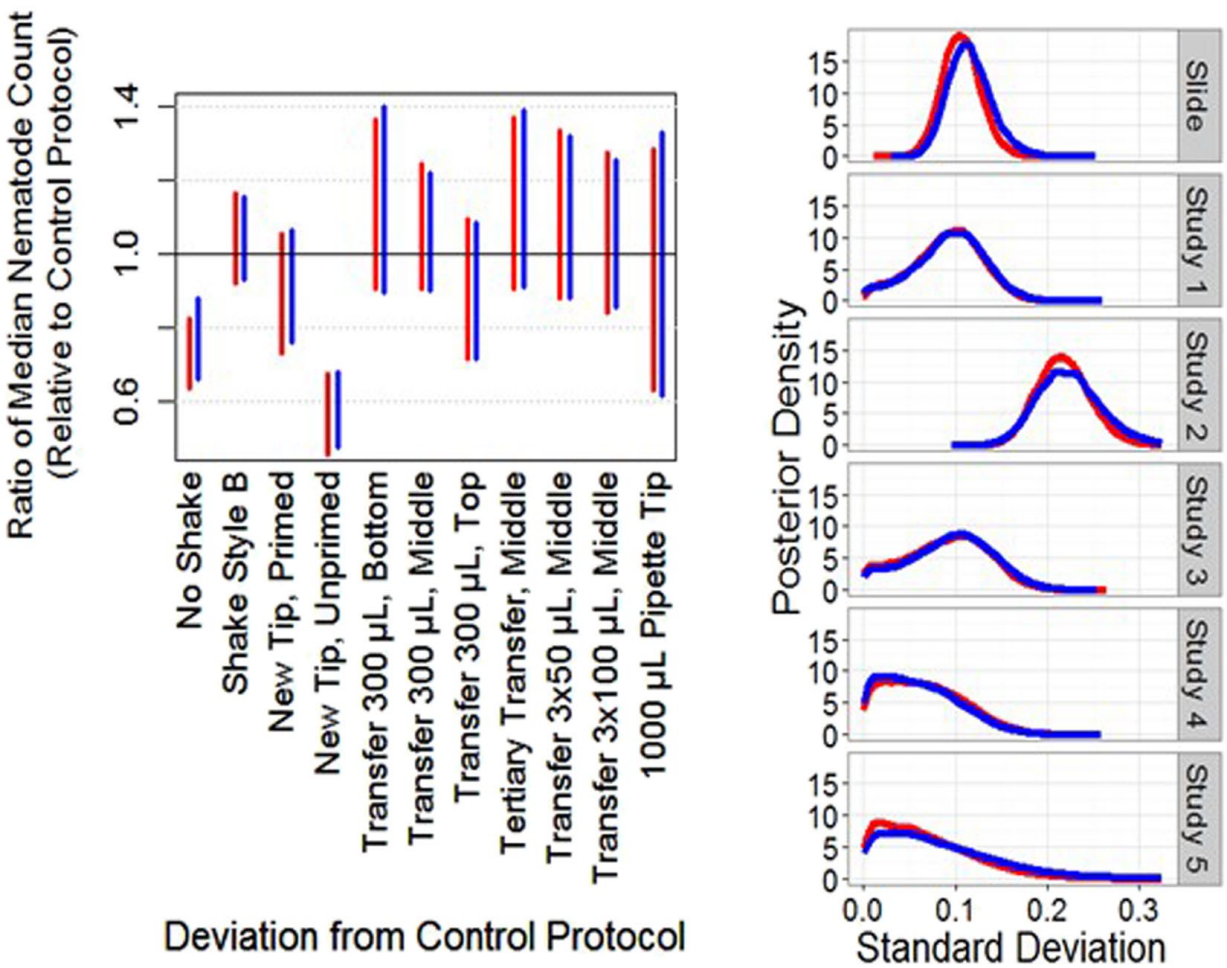
Model results for the Counting Protocol Investigation. Both panels present results from model 1 (red) and model 2 (blue). Left: The estimated impact of each modified protocol is depicted by a 95% posterior credible interval for the model parameter controlling the ratio of the median count under the modified protocol to the median count under the standard protocol. Right: Posterior density estimates for the standard deviations of random effects attributed to slide, and the random error allowing for overdispersion in each of the five studies.

Because many steps in the counting process can influence count numbers, we performed a cause-and-effect analysis to identify testable sources of bias in the general counting process (Fig. 1). An overview of the data collected as part of the counting protocol investigation is provided in Fig. 2. Counting tests designed to assess the identified potential sources of variability are described in Table 1 and Supplementary Table S1. Seven possible sources of error in nematode counting were tested in 14 different counting tests. Details on the experimental procedures are available in the Supplementary Information. The standard counting protocol outlined in the Methods section and described in detail below (10 × 2 μL aliquots extracted directly from a flask, priming a reused pipette tip, swirl style shaking) was used for all tests unless stated otherwise. Nematodes were counted in 2 μL aliquots or “dots” placed on a microscope slide in a specific sequence (Fig. 3), unless described otherwise. The counting tests included 14 one-factor-at-a-time alterations to the standard counting protocol: no shaking; using an alternative shaking style; using a primed or unprimed new tip or reusing a primed tip; transferring 300 µL of sample from the flask to a 1.7 mL microcentrifuge tube (henceforth referred to as “tube”) and extracting aliquots from the bottom, middle, or top; transferring 3 × 100 µL of sample to a tube and extracting aliquots from the middle; transferring 3 × 50 µL of sample to a tube and extracting aliquots from the middle; and using a 1000 μL pipette tip to place aliquots into a 24-well plate. Figure 4 presents 95% posterior credible intervals for parameters in statistical models 1 and 2 (used to assess model sensitivity and described in the Supplementary Information) that assess the multiplicative effect each modified protocol has on median count, compared to that for the standard counting protocol (i.e., ${\tau }_{p}$ from Equations 1 and 2; equations in the Supplementary Information). The interval corresponding to the protocol of not shaking the flask extends from 0.64 to 0.83, indicating that not shaking the sample between aliquot extractions is estimated to reduce median nematode counts by 17% to 36%. The interval corresponding to the protocol using a new, unprimed pipette tip ranged from 0.46 to 0.67, indicating that using a new, unprimed pipette tip is estimated to reduce median counts by 33% to 54% compared to a previously used and primed tip.

**Table 1:** Tests Employed to Determine Sources of Error and Best Practices for Counting Nematodes.

Source of Error Tested	Description of Test
Size exclusion	Size of tip: standard (10 × 2 μL) vs. imaging of aliquot from 1000 μL tip
Settling bias	Shaking style A vs. shaking style B
	Shaking of flask style A vs. no shaking
	Shaking of flask style B vs. no shaking
Loss during transfer	New, unprimed pipette tip vs. re-used, primed tip
	Primed pipette tip vs. not primed tip
	Aliquot from flask vs. transfer to and aliquot from tube
Sampling location bias	Top vs. middle vs. bottom of tube
	Counts from flask vs. counts from tube
Between-people variability	Operators counted the same flask with known concentration of nematodes
	Operators counted the same flask with blinded concentration of nematodes
Effects of transfer	Volume amount: 3 × 100 μL vs. 3 × 50 μL
	Number of transfers: 3 × 100 μL vs. 1 × 300 μL
Location bias	Order of dots on slide

Whether the other protocol modifications substantially influenced nematode counts was inconclusive. Uncertainty intervals corresponding to each of the remaining eight protocol modifications included 1, a value corresponding to no effect on median nematode count, and extended to values far from 1, which correspond to substantial influence on median count. For instance, according to the 95% posterior credible interval for tertiary transfer, this process may have decreased the median count by as much as 10% or increased the median count by as much as 40%, when compared to the standard process that sampled aliquots directly from the culture flask. The interval corresponding to the protocol that uses a 1000 µL pipette tip has a wider range due to the acquisition of only six counts under this protocol and use of a single control slide for comparison.

Ninety-five percent posterior credible intervals summarizing pairwise comparisons between various protocols investigating a common variability source are provided in Supplementary Tables S2 through S7. The direct comparison between extracting aliquots from the top and bottom of a test tube shown in Supplementary Table S5 may provide evidence of a settling effect in that the 95% posterior credible interval suggests the median count obtained when extracting from the bottom is between 0 and 60% greater than the median count obtained when extracting from the top.

**Figure 5:**
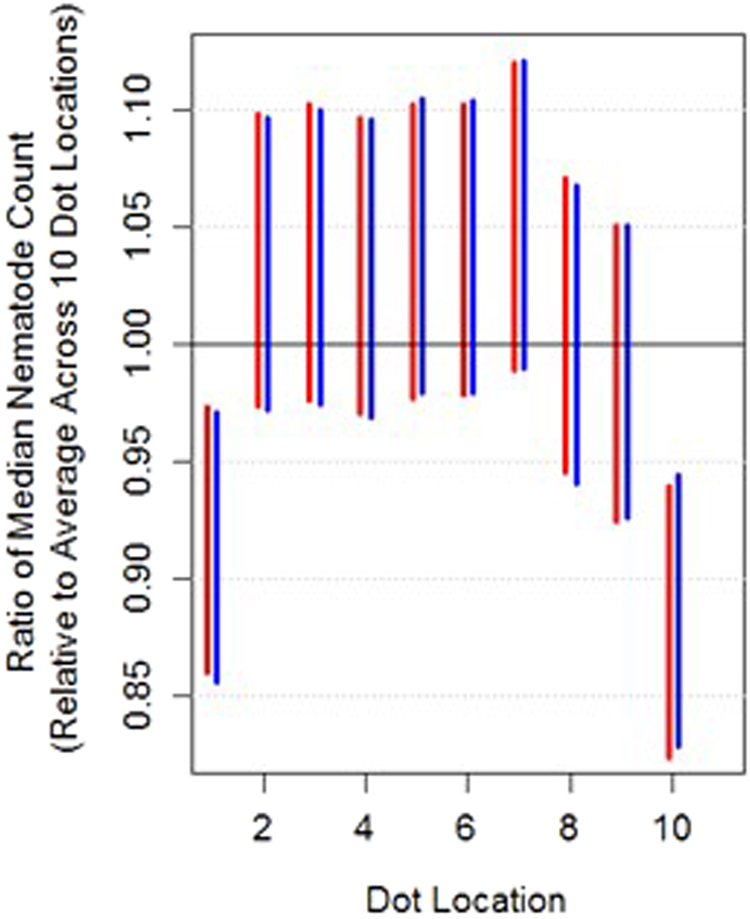
Model results for Dot Location Effects. Results from models 1 and 2 are displayed in red and blue, respectively. The 95% posterior credible intervals for the model parameter controlling the ratio between the median count for each dot location versus the average of median counts across all 10 dot locations. Results indicate a clear effect at the first and last (10^th^) positions.

We examined whether the distribution of nematode counts appeared to be affected by when and where the 2 μL dots were placed within slides. The average nematode count ranged from as low as 16.3 for spot 10 to as high as 19.8 for spot 2 across all 63 slides used in the variability experiments. Figure 5 presents 95% posterior credible intervals for the ratio of median count for each dot location versus the average of medians across all 10 dot locations (${\gamma }_{i}$ from Equations 1 and 2, in Supplementary Information). The results of this analysis suggest that dot locations 1 and 10 had a statistically significant tendency to produce lower counts compared to any of the dot locations 2 through 9. According to the models, the median count from dot location 1 is 3% to 14% lower than the average of the median counts across all 10 dot locations. The median for dot location 10 is 5% to 18% lower than the average median across all 10 dots. This surprising result was corroborated by a non-parametric sign test described in the Supplementary Information with additional information in Supplementary Table S8.

**Figure 6:**
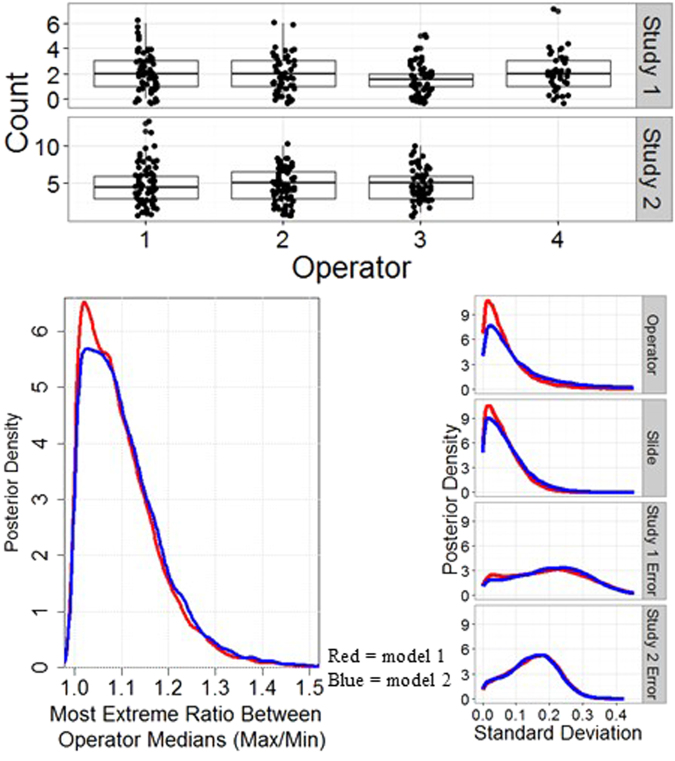
Operator-to-Operator Variability Studies. Top: Nematode count data from two experiments designed to examine the operator-to-operator variability in counts. Bottom: Both lower panels present data from model 1 (red) and model 2 (blue). Bottom Left: Posterior density estimates for the ratio between the largest median count to the smallest median count among the four participating operators. Bottom Right: Posterior density estimates for the standard deviations of random effects attributed to general population of operators, slides and the random error allowing for overdispersion in each of the two studies.

Data from the inter-operator variability studies and corresponding modeling results are shown in Fig. 6. In the bottom right panel of Fig. 6, the similarity between the posterior density plots (plot of probability, evaluated after taking into account relevant evidence related to each analysis) for the standard deviations of operator effects and slide effects, respectively, indicates that the impact of using two different operators was roughly equivalent to the impact of using two different slides. As seen in the bottom left panel of Fig. 6, the largest ratio [i.e., exp(maximum $\{{\tau }_{1},{\tau }_{2},{\tau }_{3},{\tau }_{4}\}\,-$ minimum $\{{\tau }_{1},{\tau }_{2},{\tau }_{3},{\tau }_{4}\}$), for $\tau$ as defined in Equations 3 and 4, in the Supplementary Information] among the median counts of the four operators had posterior modes of 1.03 and 1.06 under the first and second models, respectively. The larger of these two values corresponds to a 6% range in median counts across the operators. As a more conservative interpretation, the upper bounds for a one-sided 95% posterior credible interval for the largest ratio roughly corresponded to a 25% range in median counts across the four operators. Pairwise 95% posterior credible intervals for the ratio of median counts between operators [i.e., exp(${\tau }_{o}-{\tau }_{{o}^{^{\prime} }})$] are provided in Supplementary Table S9.

The results reported in this section for the protocol models and for the operator models appeared consistent across two variations of each model, respectively. Although this indicates that the stated results are not unique to one specific subjective modeling choice, further consideration of additional models not evaluated in this analysis may produce different results. Analyzing additional samples would further reduce the uncertainty underlying these assessments.

From this complete sequence of experiments, distinct effects reducing overall nematode count were observed when the sample flasks were not shaken and when pipette tips were not primed with nematode culture before counting nematodes in sample aliquots. Smaller but statistically significant effects on nematode counts include decreased nematode counts at the first and last “dot” locations on the slide. A trend for decreased nematode counts for aliquots removed from the top volumetric portion versus the bottom volumetric portion of sample containers (e.g., microcentrifuge tubes) was also seen.

### Standardized protocol for nematode counting in liquid culture

The protocol described is for 10 mL cultures grown in 25 cm^2^ tissue culture flasks. A detailed protocol and schematic (Supplementary Figure S1) can be found in the Supplemental Information; a simplified protocol follows. Modified CeHR cultures should only be opened in a sterile BSL-2 hood to ensure sterility (the milk-based medium is especially susceptible to contamination). S-basal complete cultures can be opened and passaged on a bench-top sprayed with 70% volume fraction ethanol. It is important to prime pipette tips, shake/swirl the culture flasks before sampling and vortex mix any secondary containers, e.g, microcentrifuge tubes, as described.

Place a sterile 10 μL pipette tip onto a 2 or 10 µL pipette. Shake the flask gently, taking care to not wet the cap filter, unscrew and remove the flask cap, and prime the pipette tip by pipetting the contents up and down at least four times in the nematode culture. Tips can be reused for all 10 dots in one counting replicate. Remove a 2 μL aliquot from the culture, check to make sure the sample is in the pipette tip, and transfer the sample onto a sterile, glass microscope slide at the top left corner of the slide (dot position 1, Fig. 3). After removing the aliquot, gently shake the (upright) flask before sampling again. Place dots onto the glass slide in the following order: dot 1 is placed at the top left of the slide near the slide label or edge. Dots 2 to 5 are placed to the right of each preceding dot. Dot 6 is placed on the bottom right of the slide (below dot 5). Dots 7 to 10 are placed to the left of each preceding dot.

Examine the slide using a light microscope and count nematodes at 40× magnification (4× objective and 10× eyepiece) beginning with the first dot. Count the number of nematodes of all sizes, including juveniles and adults. It is also possible to record dead nematodes, quantify nematodes of different stages and identify “bag of nematodes”, or nematode adults with internal hatched neonates (indicative of a problem with the organism and potentially with the culture). If the nematode culture is too dense to accurately count, dilute the culture as described in the Supplementary Information. Record counts and dilutions. Wipe the slide clean and spray the microscope slide with 70% volume fraction ethanol before reusing.

### Nematode population growth rates in different liquid media

**Figure 7:**
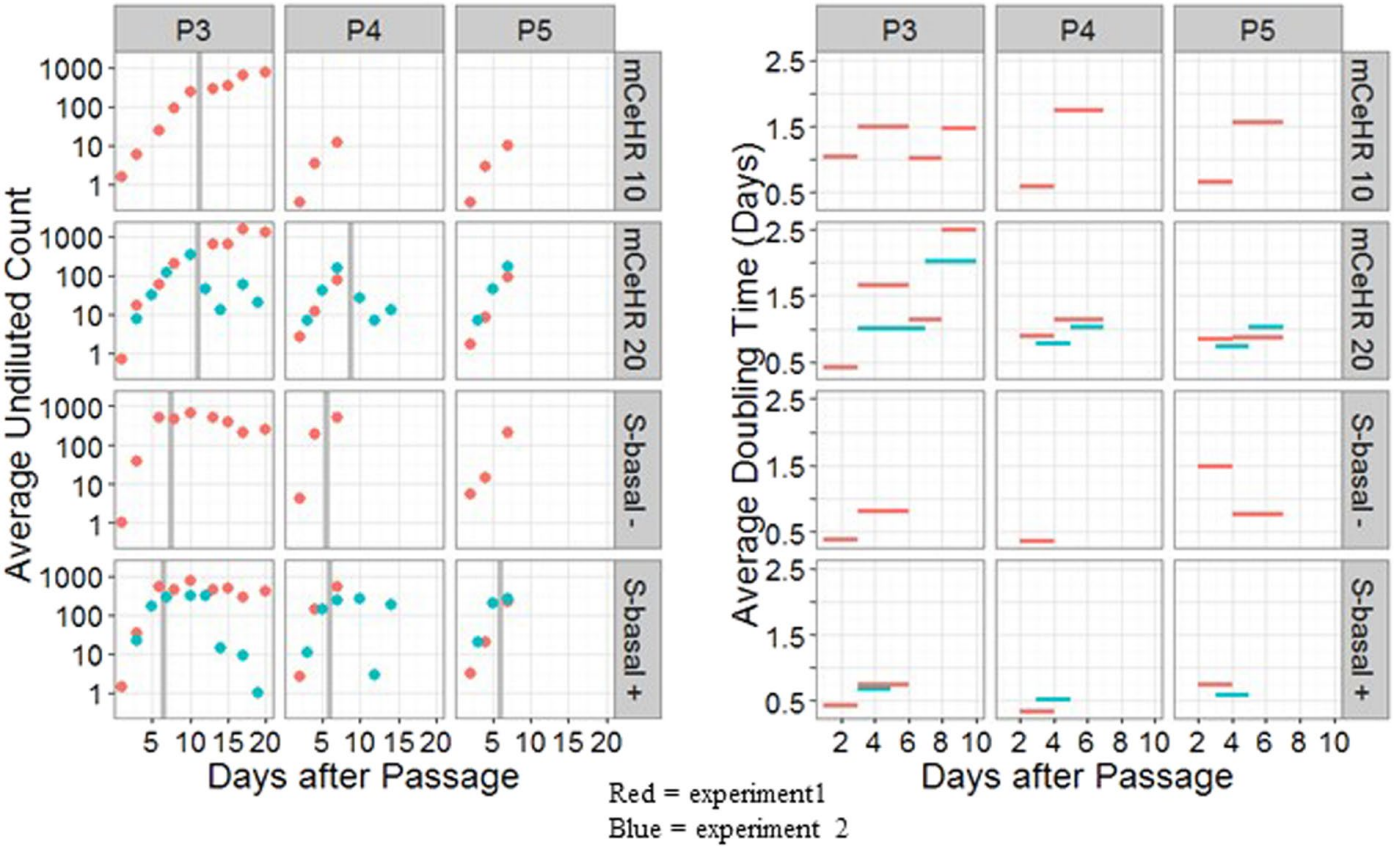
Nematode Growth Rate. Results from the first and second growth rate experiments are shown in red and blue, respectively. Results from different growth media are separated by row. Results for passages 3, 4 and 5 are separated by column. Left: Average count data, as calculated according to Equation 5 (in the Supplementary Information). Right: Estimated doubling time, as calculated according to Equation 7 (in the Supplementary Information). The vertical gray lines seen in some panels on the left convey time points at which growth rate appeared to be influenced by high nematode concentrations, as subjectively identified by the authors.

Nematode count data for passages P3, P4 and P5 across experimental conditions in both population growth experiments were analyzed to assess the average count (${{\bar{Y}}^{\ast }}_{eptd}$ from Equation 5, in SI) and exponential population growth rates (${\rm{\Delta }}{T}_{eptdd^{\prime} }$ from Equation 7, in SI), as displayed in Fig. 7. Note that the left panel of Fig. 7 displays average count using a log scale, so that counts during a phase of exponential growth with a constant rate would appear along a straight line. It was observed that as average counts approached or exceeded 100, nematode populations grew at a slower rate or even declined, which may indicate a limit to the concentration of a healthy culture. The vertical gray lines seen in some panels of the left side of Fig. 7 convey time points at which growth rate appeared to be influenced by dense nematode concentrations, as subjectively identified by the authors; count data occurring to the right of these vertical lines were not considered when computing growth rates. For each treatment condition, growth results appeared generally consistent between experiments and passages during the initial phase of population growth before becoming highly divergent at later stages.

These results may indicate an increased growth rate under S-basal conditions as compared to mCeHR conditions. In each of the nine combinations of experiment, passage, and S-basal growth conditions (either S-basal +, with tetracycline antibiotic or S-basal -, without tetracyline antibiotic), the average count exceeded 100 by day 5 (or the first counting session after day 5, in the event that operators were unable to obtain a count until after day 5). For comparison, all of the average counts under mCeHR conditions (with either 10 or 20% milk) corresponding to those same days were below 100. The population doubling times, displayed in the right half of Fig. 7, tended to be around one day or longer for mCeHR growth conditions. In 11 of 12 time intervals between adjacent counting sessions for S-basal growth conditions, the estimated doubling time was less than one day. Results generally appeared comparable between mCeHR 10 and mCeHR 20 and between S-basal + and S-basal − growth conditions.

It is difficult to decouple random noise in the estimated doubling times from possible trends of decreasing growth rates as days after passage increases. Future studies may benefit from determining population counts at least daily during initial growth phases and discontinuing once a threshold for average count has been exceeded.

### Nematode hatching rates in different liquid growth media

Throughout the population growth experiments, it was perceived that initial nematode eggs tended to hatch earlier under S-basal growth media than under mCeHR treatment, although formal data had not been collected regarding this effect. During a brief follow-up experiment, the number of days until egg hatching was evaluated for five independent replicates in each of the two nematode culturing conditions, S-basal complete and mCeHR. In all five replicates under S-basal complete conditions, the first egg hatching occurred on the fourth day. In four of the fives replicates under mCeHR treatment, the first egg hatching occurred on the fifth day, with the remaining replicate producing young on the fourth day. These data suggest that eggs in S-basal complete tend to hatch about one day faster than eggs grown in mCeHR. Applying a Fisher’s Exact test to the corresponding 2 × 2 contingency table (Supplementary Table S10) produced a two-sided p-value of 0.048, indicating moderate statistical significance of the observed difference.

## Discussion

The nematode *C. elegans* is an important model organism widely used in diverse fields of study. Nematodes in liquid culture are convenient for different types of experimental procedures and are highly amenable for high-throughput studies. Furthermore, nematodes in liquid culture typically encounter less mechanical stress than nematodes traditionally grown on plates^35^. However, when conducting research with nematodes in liquid culture, it is important to establish “fixed” or standardized methods among different researchers in order to facilitate reproducible results^35^. The use of standardized methods, such as methods regularly established by the International Organization for Standardization (ISO) and the United States Environmental Protection Agency (US EPA), is the norm for the proper conduct of research across many scientific fields and in industry. A reliable counting protocol for nematodes in liquid culture is a necessary tool for reliable measurements. In this work, we describe a rigorously standardized, general counting protocol for nematodes in liquid culture, in which we found 14 testable potential sources of error. Of those, three had significant effects on nematode counts: (1) shaking of the culture, (2) priming of pipette tips and (3) sampling location. Using our standardized protocol for counting nematodes in liquid culture, we observed that nematode population growth rates may be greater in S-basal versus mCeHR medium. We separately observed that nematode eggs in S-basal complete media hatched approximately 1 d before those in mCeHR.

The 14 experiments we conducted to test for seven types of variability in the nematode counting method allowed us to better understand potential influences within the counting process. From this complete sequence of experiments, distinct effects reducing overall nematode count were observed when the sample flasks were not shaken and when pipette tips were not primed with nematode culture before counting nematodes in sample aliquots. Smaller but statistically significant effects on nematode counts include decreased nematode counts at the first and last “dot” locations on the slide. A trend for decreased nematode counts for aliquots removed from the top volumetric portion versus the bottom volumetric portion of sample containers (e.g., microcentrifuge tubes) was also seen, which is likely due to settling of nematodes in liquid culture (e.g., there are more nematodes at the bottom of the tube than at the top). This work represents the most rigorously standardized nematode counting method published to date.

It was surprising to find such low variability in the nematode counts performed by different operators (i.e., person-to-person bias) because standardization of sample preparation processes in biology and molecular biology that require human interactions is notoriously difficult^15,36,37^. However, as our laboratory is housed in a metrology institution, we pay particularly close attention to protocol detail in general, and regularly refine processes to maximize reproducibility. It should be noted that counts were performed by people with different educational and experiential backgrounds (from undergraduate interns to postdoctoral research fellows).

Potential bias caused by ordering of dot placement on the slide is another surprising result, although this result was derived from measurements taken by one single analyst and thus cannot be generalized to a broader population. When properly sampled, e.g. shaken culture and primed tip, there is no obvious reason why the number 1 (placed first) and number 10 (placed last) dots would be significantly lower in nematode counts than the other eight dot locations. We have visually observed that the first dot can sometimes look smaller than the remaining nine dots, and in the past attributed this phenomenon to an interaction between the pipette and the dry microscope slide. However, we lack convincing explanations for the observed differences, when the dots are sampled and placed on the slide using the same procedure.

Results regarding shaking the culture and priming pipette tips were not surprising; nematodes are known to settle in liquid culture and will stick to polymer materials such as pipette tips^38,39^. Shaking the culture distributes the nematodes more evenly, while priming the pipette tips by wetting with culture medium prevents the nematodes from sticking.

While we believe that the described nematode counting method represents the most rigorous, reproducible counting method currently available, other uncontrolled factors may still limit the accuracy of nematode counts in liquid culture. For example, counts performed on cultures containing gravid adults will necessarily change dramatically over time as eggs are hatched and larvae are formed.

An important area of research that regularly relies on nematode counts is the study of lifespan extension through calorie restriction. Interestingly, published references in this research area typically do not include detailed methodology for important experimental procedures nor do the references include details regarding optimization of the methodology. Reproducing the research in these references may prove to be difficult. Houthoofd and Vanfleteren (2006) review different methods for dietary restriction and lifespan measurement in *C. elegans*. However, the authors provide no details on how animals are transferred, counted or grown. This makes quantitative or qualitative comparison of reported results practically impossible^25^. The research by Solis and Petrascheck (2011) on the development of a 96-well plate-based assay for chemical screening provides detailed methodology, but does not discuss how (or if) method optimization was performed^26^. In the work by Panowski *et al*.^24^, two supplemental files and two additional references had to be downloaded in order to compile all of the described methodology^24^, but it remains unclear whether the summary methodology is sufficient to allow adequate reproduction of the described research. All of these reports are interesting, informative, impactful and published in well regarded journals. However, inconsistent methodological detail may potentially pose limitations on experimental reproducibility. Our counting protocol promises to help advance the reproducibility of *C. elegans* research by providing a rigorous and novel analytical and statistical assessment of common procedures and experimental steps that many researchers may take for granted.

The population growth rate experiments appear to suggest that cultures in S-basal media tend to expand more rapidly than those in mCeHR media. The growth of nematodes is dependent on food availability and other environmental factors that can be highly species-specific. For example, three species of bacterial-feeding nematodes, *Diplolaimelloides oschei*, *Diplolaimelloides meyli* and *Pellioditis marina*, which co-occur on macrophyte detritus in the Westerschelde Estuary in the Netherlands, grow at different rates in the same environment and will grow at different rates than sibling, same-species colonies in different environments^40^. L2 larval nematodes grown under bacterial dietary restriction take 24 h longer to become gravid adults at 20 °C, with an increased average lifespan (12 d), compared to 5 d for nematodes fed *ad libitum*^39^. In our brief follow-on study, it appeared that eggs inoculated in S-basal complete cultures hatched roughly one day sooner than those inoculated in mCeHR cultures. Although this result was found to be marginal in terms of statistical significance, it is a very coarse estimate and could be refined by more frequent assessments of whether hatching has occurred. It is not clear what caused the apparent difference in hatching rates, but other researchers have showed slower growth of nematodes in axenic medium in comparison to nematodes grown on NGM plates^41^. Time of nematode development from an egg can also vary depending on incubation temperature^42–44^. Samuel *et al*. show that nematodes grown in mCeHR initially grow more slowly than those on NGM plates, requiring 7 to 10 d to become gravid^34^. Over time, the time decreased to 4 d, similar to the 3.5 d required for nematodes grown on plates. However, Samuel *et al*. do not report summary data on growth rates or statistical analyses. Furthermore, it is preferable to compare effects of different conditions on growth from data generated in the same lab, at the same time; in the present study, the growth rate of sister nematode cultures in two different liquid medium were compared, thus removing lab-to-lab variability.

This work accomplished three goals in the standardization of *C*. *elegans* liquid culture methods. First, we determined possible sources of error when counting nematodes in liquid culture. Second, we minimized variability when counting nematodes in liquid culture and developed, tested and standardized a robust, reproducible nematode counting protocol. Finally, we used our standardized nematode counting protocol to assess population growth rates of nematodes in different liquid growth media and detected differences in hatching times of nematodes hatched in biologically distinct media. By systematically identifying and methodically testing potential sources of error, we established a high-quality, easy and reproducible method that will be useful and adaptable to any laboratory utilizing *C. elegans* in liquid culture.

## Methods

### *C. elegans* and *E. coli* OP50 culturing

*C. elegans* N2 wild-type isolates and *E*. *coli* OP50 (OP50) were obtained on agar plates from the Caenorhabditis Genetics Center (University of Minnesota). Detailed methods and recipes for nematode and OP50 culture are described in the SI. After establishing nematode colonies on Nematode Growth Medium plates with OP50 as a food source, nematodes were collected and bleached^34^ to obtain a sterile solution of nematode eggs. Eggs were re-suspended in mCeHR^34^ or S-basal complete nematode growth media. mCeHR was supplemented with 10% or 20% volume fraction fat-free milk (ultra-pasteurized; opened in sterile hood only) + 100 μg/mL tetracycline hydrochloride (Sigma). S-basal complete was supplemented with 10% volume fraction OP50 suspension with or without 1% Penicillin-Streptomycin-Amphotericin B and 0.5% Amphotericin B (MP Biomedicals). It is important to note, culture flasks must be vented for adequate air supply; when swirling/shaking flasks, take care to not wet the vent with culture medium so as to not contaminate cultures.

### Examining the influence of experimental protocol on nematode counts

Counting nematodes in liquid culture poses numerous experimental challenges. The nematodes move sporadically, settle to the bottom of tissue culture flasks or microcentrifuge tubes, and can stick to pipettes tips. Preliminary results indicated variability can be caused by changes in protocol such as differences in pipette tip volumes or changes in operator handling procedures. We therefore conducted a cause-and-effect analysis to identify sources of variability in the nematode counting process (Fig. 1). Sensitivity testing was then used to assess which sources of variability have significant impacts on the overall process. Cause-and-effect diagrams in combination with sensitivity analysis facilitate the adoption of appropriate process controls/measurements to ensure that the processes are performing as expected^13,45^.

S-basal complete nematode cultures were used for protocol development. The general protocol for counting nematodes is to hold the flask by the neck, briefly shake or swirl the flask (without wetting the vent cap) and then transfer 10 × 2 μL aliquots from the flask onto a standard glass microscope slide, with gentle shaking of the flask between each aliquot or “dot.” A volume of 2 μL is used for ease of nematode recognition: the entire dot can be visualized under a light microscope at 40× magnification (4× objective and 10× eyepiece) without moving the glass slide, and 10 × 2 μL dots fit well on a standard glass microscope slide. Nematodes are counted manually/visually; the process is not automated. Dots are always counted from position 1 to position 10 (discussed in detail, below). Using additional dots per slide provides diminishing returns in terms of reducing uncertainty, as standard errors generally scale at 1/$\sqrt{n},$ where $n$ is the number of independent replicates.

All growth containers were 10 mL, 25 cm^2^ tissue culture flasks. Secondary vessels/containers were 1.7 mL graduated flat top microcentrifuge tubes (Avant). Pipette tips were sterile (10, 20, 200 or 1000) µL Eppendorf epT.I.P.S. Pipettes were calibrated (10, 20, 200 or 1000) µL Eppendorf Pipette Plus models. Each replicate included 10 × 2 μL dots placed on a glass microscope slide in sequence from dot location 1 to dot location 10, as in Fig. 3, except when indicated otherwise. Pipettes used for counting were always primed by pipetting up and down in growth culture four times, except where indicated. Three or more replicates were performed per test (for a total of at least 30 dots per condition). Pipettes and tips were chosen following a pilot study where the accuracy of pipette and tip volume was measured by measuring the mass of pipetted water (data not shown). Samples in microcentrifuge tubes were vortex mixed (Vortex Genie-2, Scientific Industries) on medium speed for 4 s immediately before counting, but were not vortex mixed between pipetting the individual dots.

We identified seven aspects of the counting protocol whose influence on nematode count could impact the counting results: size exclusion from tips; settling of nematodes; loss to tips or secondary vessels during transfer; sampling location bias in flasks and tubes; between-people variability; effects of transfer of different volumes or numbers of aliquots; and location bias or spatial/temporal order of sample aliquot placement on slides. We then conducted experiments to examine the influence of each variable. The counting tests are described in Table 1 and Fig. 1. Detailed experimental procedures for each counting test are detailed in Supplementary Table S1 and a schematic is provided in Supplementary Figure 1.

### Determination of nematode population growth rates in different liquid growth media

Two sets of experiments were conducted to measure the nematode population growth rate in S-basal complete or mCeHR liquid growth media. For the first set of experiments, nematodes grown on NGM plates were bleached to obtain a sterile solution of eggs. Eggs were aliquoted into S-basal complete with 10% volume fraction OP50 suspension with or without tetracycline antibiotic (denoted as S-basal+ and S-basal−, respectively) or into mCeHR with 10% or 20% volume fraction fat-free milk (denoted as mCeHR 10% or mCeHR 20%, respectively). These experiments were designed to assess whether the presence of antibiotics affected the OP50 food source in the S-basal complete cultures or if nematodes grew better with an increased concentration of milk. All experiments with mCeHR cultures were performed in a sterile Biosafety Level 2 cabinet with the cabinet light turned off. Experiments with S-basal complete cultures were performed in a nematode-only designated area of a laboratory benchtop sterilized with 70% (volume fraction) ethanol and a disinfectant spray.

On Day 0, nematode cultures were bleached to obtain sterile egg solutions. Fifty mL of each of the four media types was prepared and aliquoted into three biological replicates with 10 mL medium per condition (mCeHR 10%, mCeHR 20%, S-basal+, S-basal−). A 6.6% volume fraction of nematode eggs suspended in sterile water was added to each biological replicate. This was considered Passage 1 (P1). Throughout the first experiment, each biological replicate was monitored by a designated operator. That is, three operators each monitored one biological replicate per treatment condition. Monitoring and subsequent counting were performed by highly trained (post-doctoral) and novice (undergraduate) operators. All nematode cultures were incubated in the dark at 21 °C in an incubator; mCeHR and S-basal complete cultures were housed on separate shelves in the same incubator. Cultures were visually checked by eye for contamination 5 d per week (contamination is evidenced by clumping of OP50 in S-basal cultures or coagulation of milk in mCeHR cultures). Nematodes were passaged (split; divided into fresh medium) weekly or bi-weekly to acclimatize the nematodes to liquid culture. Passaging was performed by one operator for all cultures at the same time. For the first two passages, on Day 2 and Day 8, cultures were counted and approximately equal numbers of nematodes were added to each new culture: 1000 nematodes for P2 and 250 nematodes for P3. With the start of P3, counting was performed 3 d per week (Monday, Wednesday and Friday) to inform growth rate estimates. Culture passages on Day 14 (P4) and Day 21 (P5) were performed by transferring 50 μL of nematode culture from the previous passage into the new culture; equal volumes of culture were transferred instead of equal numbers of nematodes as a way to increase potential differences in nematode counts (a culture with more added nematodes will increase faster in numbers than a culture with fewer added nematodes). Nematode counts were recorded for further statistical analyses.

For the second set of growth rate experiments, the bleaching, culturing and passaging processes were repeated with a few modifications – only mCeHR 20% and S-basal+ complete were tested, and the number of replicates was increased to five biological replicates per condition. As with the first experiment, each biological replicate was monitored by a designated operator, and five operators each monitored one biological replicate per treatment condition. P3, P4 and P5 were counted for the entire duration of the study, instead of for only one week. All other methods were consistent with the first set of experiments: cultures were started from bleached nematode stocks, passaged twice at 1000 and 250 nematodes per culture (P2 and P3), then passaged twice at 50 μL per culture (P4 and P5), and counted three times per week.

### Statistical analyses

The statistical software R^46^ was used with the following packages: ggplot2^47^, tidyr^48^, Dplyr^49^, rjags^50^ and R2WinBUGS^51^. Statistical analysis typically consisted of a generalized mixed effects model with an over-dispersed Poisson distribution. Detailed descriptions of the modeling methods are provided in the Supplementary Information.

### Data availability statement

All data generated or analyzed during this study are included in this published article (and its Supplementary Information files).

### NIST Disclaimer

Certain commercial equipment, instruments and materials are identified in this paper to specify an experimental procedure as completely as possible. In no case does the identification of particular equipment or materials imply a recommendation or endorsement by the National Institute of Standards and Technology nor does it imply that the materials, instruments, or equipment are necessarily the best available for the purpose.

## Electronic supplementary material

Supplementary Information
