# Supplementary material for: Counting Caenorhabditis elegans: Protocol Optimization and Applications for Population Growth and Toxicity Studies in Liquid Medium
Source: Sci Rep. 2018 Jan 17;8:904. doi: 10.1038/s41598-018-19187-3 (PMC5772475; doi:10.1038/s41598-018-19187-3)
Supplement: Supplementary file 1 — Supplementary Information [file 41598_2018_19187_MOESM1_ESM.pdf]

Supplementary Information

Counting *Caenorhabditis elegans*: Protocol Optimization and Applications for Population Growth and Toxicity Studies in Liquid Medium

Leona D. Scanlan[1a*†], Steven P. Lund[2†], Sanem H. Coskun[3#], Shannon K. Hanna[3], Monique E. Johnson[4], Christopher M. Sims[3], Karina Brignoni[1], Patricia Lapasset[4], Elijah J. Petersen[3], John T. Elliott[3] and Bryant C. Nelson[3*]

[1]National Institute of Standards and Technology, Material Measurement Laboratory – Biomolecular Measurements Division, 100 Bureau Drive, Gaithersburg, MD 20899, United States

[2]National Institute of Standards and Technology, Information Technology Laboratory – Statistical Engineering Division, 100 Bureau Drive, Gaithersburg, MD 20899, United States

[3]National Institute of Standards and Technology, Material Measurement Laboratory – Biosystems and Biomaterials Division, 100 Bureau Drive, Gaithersburg, MD 20899, United States

[4]National Institute of Standards and Technology, Material Measurement Laboratory – Chemical Sciences Division, 100 Bureau Drive, Gaithersburg, MD 20899, United States

[#]Department of Pharmacognosy, Faculty of Pharmacy, Gazi University, 06330 Ankara, Turkey

[a]Present address
California EPA Department of Pesticide Regulation, Sacramento, CA 95814, United States

[†]These authors contributed equally to this work.

* To whom correspondence should be addressed:

bryant.nelson@nist.gov, 301-975-2517 (ph); 301-975-8542 (fax)
scanlan.leona@gmail.com, 916-445-4207 (ph); 916-324-3506 (fax)

Methods

Nematode and *E. coli* OP50 Culture Details

E. coli OP50 was cultured in Lennox L. Broth Base (Invitrogen) for 17 h at 37 °C (with shaking), washed twice (centrifugation at a fixed angle at 2000 x *g* for 10 min) and re-suspended in *C. elegans* M9 growth buffer (medium composition described below). The OP50 is stored at 4 °C for up to one week (microbes start to die and aggregate after one week). A volume of 100 µL OP50 suspension was spread onto agar Nematode Growth Medium (NGM, recipe in SI) plates and incubated overnight at room temperature (approximately 24 °C). "Chunks" of *C. elegans* on agar were transferred from the stock plate to NGM OP50 plates and incubated in a dark incubator at 21 °C for a period of 5 d to 7 d. Once nematodes consumed the *E. coli* and cleared the plates, chunks of the NGM nematode culture were again transferred to fresh OP50 plates. The "chunking" process was repeated 4 to 5 times to establish healthy, gravid nematode populations. Nematodes were monitored with a light microscope at 40X magnification. After they had consumed most of the OP50 lawn, 5 mL M9 was added to the place and a sterile bacteria spreader was used to gently dislodge the eggs; egg and medium slurry was pipetted into a centrifuge tube and bleached [1] to obtain a sterile solution of nematode eggs. Eggs were washed twice with sterile water and re-suspended in the two different nematode growth media. mCeHR[1](recipe in SI) was supplemented with 10 % or 20 % volume fraction fat-free milk (ultra-pasteurized; opened in sterile hood only) + 100 µg/mL tetracycline hydrochloride (Sigma). S-basal complete (SI) was supplemented with 10 % volume fraction OP50 suspension with or without 1 % Penicillin-Streptomycin-Amphotericin B and 0.5 % Amphotericin B (MP Biomedicals). Buffer and medium protocols are described in detail in the SI. It is important to note, culture flasks must be vented for adequate air supply; when swirling/shaking flasks, take care to not wet the vent with culture medium so as to not contaminate cultures.

Statistical Analyses

Variability studies

Data from the counting protocol experiments were analyzed using a generalized mixed effects model, described below. Let Y_{dpsi} denote the observed count for dot position i on slide s for protocol p on the sample prepared for experiments conducted on day d. Y_{dpsi} was modeled in an overdispersed Poisson distribution with conditional mean:

$$\lambda_{dpsi}|\alpha_{s(dp)}, \epsilon_{dpsi} = \mu_d * \mathrm{e}^{(\tau_p + k_p + \gamma_i + \alpha_{s(dp)} + \epsilon_{dpsi})} \qquad \text{(Equation 1)}$$

[i.e., $Y_{dpsi}|\lambda_{dpsi} \sim \text{Poisson}(\lambda_{dpsi})$], where μ_d denotes the median nematode concentration of the sample prepared for experiments conducted on day d as determined via the standard counting protocol in dot position 1; τ_p denotes the log ratio of the median concentration obtained via counting protocol p to that of the standard counting protocol; k_p is fixed as log(500/150) for the protocol using a 1000 µL pipette tip and is fixed to 0 for all other counting protocols in order to account for known differences in aliquot volume and concentration [the large pipette tip protocol used 1000 µL aliquots with a dilution factor of 150; all other protocols used undiluted aliquots of 2 µL: (1000/2)/150]; γ_i represents the log ratio of the median concentration found in dot location i to the average of the median concentrations across all ten locations (parameterized such that $\sum_{i=1}^{10} \gamma_i = 0$); $\alpha_{s(dp)}$ denotes a random effect influencing the concentration of all dots on slide s (nested within each combination of day and counting protocol) by a multiplicative factor of $\exp(\alpha_{s(dp)})$; and ϵ_{dpsi} is a random error term unique to a single dot allowing for overdispersion among observed counts. Under the described model, the median count for dot location i under counting protocol p for the study conducted on day d after integrating across the distributions of random effects $\alpha_{s(dp)}$ and ϵ_{dpsi} is given by the following function of fixed effects:

$$\lambda_{dpi}^{*} = \mu_d * \mathrm{e}^{(\tau_p + k_p + \gamma_i)} \qquad \text{(Equation 2)}$$

The simplicity of this expression facilitates easy interpretation and presentation, and for this reason modeling results are discussed in terms of median counts.

In the first implementation of the model described above (henceforth referred to as "protocol model 1"), random effects attributed to the slide were modeled as independently following a normal distribution with mean 0 and standard deviation σ_α [i.e., $\alpha_{s(dp)}$~normal(0,σ_α)]. Random error terms for counts occurring on day d were modeled as independently following a normal distribution with mean 0 and standard deviation σ_d [*i.e.*, ϵ_{dpsi}~normal(0, σ_d)], allowing the modeled degree of overdispersion to vary day to day. The median nematode concentration as observed under the standard counting protocol for day d (μ_d) was assigned a uniform "prior" from 0 to 40 nematodes per dot. For clarity, a prior is a Bayesian probability distribution for a quantity (*i.e.* number of nematodes per dot) that is not supported by defined experimental observations (data). The dot location effects were assigned independent normal prior distributions with mean 0 and standard deviation 0.5, and were normalized such that $\sum_{i=1}^{10} \gamma_i = 0$. The prior assigned to the effect of the counting protocol p (τ_p) was a normal distribution with mean 0 and standard deviation 4. The standard deviations of random slide effects and the random error terms were assigned independent exponential prior distributions with mean 0.5 [i.e., σ_α ~ exponential(0.5) and σ_d ~ exponential(0.5)].

In the second implementation of the model (henceforth referred to as "protocol model 2"), random effects for slide were modeled as independently following a double exponential distribution with mean 0 and standard deviation σ_α [i.e., $\alpha_{s(dp)}$~double exponential(0,σ_α)]. Random error terms for counts occurring on day d were modeled as independently following a double exponential distribution with mean 0 and standard deviation σ_d [i.e., ϵ_{dpsi}~ double exponential $(0, \sigma_d)$], allowing the modeled degree of overdispersion to vary day to day. The median nematode concentration as observed under the standard counting protocol for day d (μ_d) was assigned a uniform prior from 0 to 40 nematodes per dot. The prior assigned to the effect of counting protocol p (τ_p) was a uniform distribution from -5 to 5. The dot location

effects were assigned independent uniform prior distributions from -0.5 to 0.5, and were normalized such that $\sum_{i=1}^{10} \gamma_i = 0$. The standard deviations of random slide effects and the random error terms were assigned independent uniform prior distributions from 0 to 1 [i.e., σ_α and σ_d~ uniform(0,1)].

A separate analysis was conducted on data obtained from two inter-operator variability studies. Let Y_{1osi} denote the observed count for the dot at position i on slide s for operator o in the first experiment, and let Y_{2oi} denote the observed count from replicate i for operator o in the second experiment (for which no slide information or dot position was reported). The observed counts Y_{1osi} and Y_{2oi} were modeled as following an overdispersed Poisson distribution with conditional mean

$$\lambda_{1osi} = \mu_1 * \mathrm{e}^{(\tau_o + \gamma_i + \alpha_{s(o)} + \epsilon_{1osi})} \qquad \text{(Equation 3)}$$

and

$$\lambda_{2oi} = \mu_2 * \mathrm{e}^{(\tau_o + \epsilon_{2oi})}, \qquad \text{(Equation 4)}$$

respectively, where μ_e denotes the median nematode concentration of the sample prepared for inter-operator variability study e (e = 1, 2) averaged across all ten dot locations; τ_o denotes a random effect influencing the distribution of all counts conducted by operator o by a multiplicative factor of exp(τ_o); γ_i represents the log ratio of the median concentration found in dot location i to the average of the median concentrations across all ten locations (parameterized such that $\sum_{i=1}^{10} \gamma_i = 0$; these effects were only included when modeling data from the first inter-operator variability study); $\alpha_{s(o)}$ denotes a random effect influencing the concentration of all dots on slide s (nested within each operator) by a multiplicative factor of exp($\alpha_{s(o)}$) (these effects were only included when modeling data from the first inter-operator variability study); and ϵ_{1osi} and ϵ_{2oi} are random error terms unique to a single dot allowing for overdispersion among observed counts.

In the first implementation of the model for inter-operator variability data (henceforth referred to as "operator model 1"), random effects attributed to operator and slide were modeled as following a normal

distributions with means 0 and standard deviations σ_τ and σ_α, respectively [i.e., τ_o~normal(0,σ_τ) and $\alpha_{s(o)}$~normal(0,σ_α)]. Random error terms for counts occurring in experiment e were modeled as following a normal distribution with mean 0 and standard deviation σ_e [i.e., ϵ_{1osi}~normal(0, σ_1) and ϵ_{2oi}~normal(0, σ_2)], allowing the modeled degree of overdispersion to vary between the two inter-operator variability studies. The median nematode concentrations (count per 2 µL) for each experiment (μ_1 and μ_2) were assigned uniform priors from 0 to 40 nematodes per dot. Location effects Exponential distributions with mean 0.5 were assigned as priors for the standard deviations of operator effects (σ_τ), location effects, slide effects (σ_α), and measurement error (σ_1 and σ_2).

In the second implementation (henceforth referred to as "operator model 2"), random effects attributed to operator and slide were modeled as following double exponential distributions with means 0 and standard deviations σ_τ and σ_α, respectively [i.e., τ_o~double exponential (0,σ_τ) and $\alpha_{s(o)}$~ double exponential (0,σ_α)]. Random error terms for counts occurring in experiment e were modeled as following double exponential distributions with mean 0 and standard deviation σ_e [i.e., ϵ_{1osi}~ double exponential (0, σ_1) and ϵ_{2oi}~ double exponential (0, σ_2)], allowing the modeled degree of overdispersion to vary between the two inter-operator variability studies. The median nematode concentrations (count per 2 µL) for each experiment (μ_1 and μ_2) were assigned uniform priors from 0 to 40 nematodes per dot. Uniform distributions from 0 to 1 were independently assigned as priors for the standard deviations of operator effects (σ_τ), slide effects (σ_α), and measurement error (σ_1 and σ_2).

For each model described above, 200,000 Markov chain Monte Carlo (MCMC) iterations were executed, recording parameter values from every 50th iteration, following a burn-in of 30,000 iterations for each of 5 independent chains. Posterior distributions for quantities of interest are based on the 40,000 sampled sets of model parameter values. Ninety-five percent posterior credible intervals (or, the Bayesian analog of a 95% confidence interval) were approximated by the 2.5% and 97.5% quantiles for the parameter of interest, as computed from the collection of 40,000 recorded MCMC simulation iterations.

Nonparametric analysis of dot location effects

We considered the set of nematode counts across the collection of 63 slides analyzed during the variability source examination phase where the first dot was placed in spot 1 (but spanning various settings of the other controlled factors). To examine whether the observed variability between spots was statistically significant, the following leave-one-out comparison was conducted. For each slide, we determined whether the count from spot 1 was greater than, less than or equal to the collective average count from the other 9 spots on the same slide. The results across all 63 slides were evaluated using a sign test, which only requires the assumption that the observed counts are independent from one slide to another. The same process was repeated with interest focusing on each of the 10 spots in turn. The results of this test, summarized in **Table S8**, suggest that the average counts obtained from spots 1 and 10 had a statistically significant tendency to be lower than the average count of the other spots. The sign test did not facilitate the estimation of the effect size in terms of influence on median number of nematodes.

Growth rate experiments

Nematode count data from the two population growth experiments were analyzed from the perspective of exponential population growth rate and absolute count. Let $Y_{eptodsi}$ denote the count recorded under experiment e for passage p of treatment condition t observed by operator o on day d in slide s at dot position i. The ten counts occurring on a common slide were summarized by the mean $\bar{Y}_{eptods} = \frac{1}{10}\sum_{i=1}^{10} Y_{eptodsi}$ and standard deviation $sd_{etodps} = \sqrt{\frac{1}{9}\sum_{i=1}^{10}\left(Y_{eptodsi} - \bar{Y}_{eptods}\right)^2}$. Various dilution fractions were used throughout the experiment to facilitate easier counting. Let DF_{eptods} denote the dilution fraction applied in experiment e for passage p of treatment condition t observed by operator o on day d. Average counts were normalized to account for variation in dilution fraction, $\bar{Y}'_{eptods} =$

$\bar{Y}_{eptods}/DF_{eptods}$. For each combination of experiment, passage, treatment and day, a weighted average normalized count (henceforth referred to as an average count) was computed as

$$\bar{Y}^*{}_{eptd} = \frac{\sum_{s,o} {\bar{Y}'{}_{eptods}}\big/{DF^2_{eptods}}}{\sum_{s,o} {1}\big/{DF^2_{eptods}}}, \qquad \text{(Equation 5)}$$

so that normalized counts from slides with greater dilution fraction were less influential.

The average counts described above were used to estimate growth rates in the following manner. The population growth rate exhibited in experiment e for passage p of treatment condition t between consecutive counting sessions occurring on days d and d' was characterized by the following equation:

$$G_{eptdd'} = \frac{log(\bar{Y}^*{}_{eptd'}) - log(\bar{Y}^*{}_{eptd})}{d' - d}. \qquad \text{(Equation 6)}$$

The resulting growth rate was translated into the corresponding time required for a population to double in size according to:

$$\Delta T_{eptdd'} = \frac{\log(2)}{G_{eptdd'}}, \qquad \text{(Equation 7)}$$

where $\Delta T_{eptdd'}$ provides an estimate of the number of days required for population number to double in experiment e for passage p of treatment condition t, according to the average growth rate observed between consecutive counting sessions occurring on days d and d'.

Analysis of the growth experiment data is predominantly exploratory due to the longitudinal nature of population growth observations and the relatively small number of experimentally independent replicates. In such instances, the results of a model-focused analysis are likely to be heavily influenced by modeling choices and assumptions, rather than information present in the data itself.

Media and Buffers

M9 Growth Buffer

For 1 L of M9 buffer:

- Autoclave KH_2PO_4, Na_2HPO_4 and NaCl in 1 L MilliQ water
- Cool, and add $MgSO_4$.

M9 growth buffer	
amount	reagent
3 g	KH_2PO_4
6 g	Na_2HPO_4
5 g	NaCl
1 mL	1 M $MgSO_4$

Nematode Growth Medium (NGM) agar plates

For 1 L agar:

- Autoclave NaCl, agar and bacto-peptone in 1 L MilliQ water.
- Cool in a 55 °C water bath for 15 minutes.
- Add $CaCl_2$, $MgSO_4$, cholesterol and KPO_4 buffer; swirl reagents to mix.
- Pour into sterile petri dishes.
- Let cool and store inverted at 4 °C.

Nematode Growth Medium	
amount	reagent
3 g	NaCL
17 g	Agar (molecular genetics grade)
2.5 g	Bacto-peptone
1 mL	1 M $CaCl_2$
1 mL	5 mg/mL cholesterol in EtOH
25 mL	1 M KPO_4 buffer (recipe below)

Potassium Phosphate (KPO_4) Buffer

For 1 L buffer:

- Make 1 M solutions of both KH_2PO_4 and K_2HPO_4 with MilliQ water.
- Add 132 mL K_2HPO_4 and 868 mL KH_2PO_4 to a clean bottle.
- Autoclave and sterile filter.

S-basal Nematode Growth Medium

To make 1L S-basal:

- Add NaCl, K_2HPO_4 and KH_2PO_4 to 0.5 L MilliQ water; fill to 1 L.
- Autoclave.
- Store at 4 °C.

S-basal growth medium	
amount	reagent
5.9 g	NaCL
1 g	K_2HPO_4
6 g	K_2HPO_4

S-basal complete

To make 0.5 L S-basal complete:

- Add potassium citrate solution, trace metals solution, $MgSO_4$, $CaCl_2$, penicillin-streptomycin, amphotericin B to 500 mL S-basal.
- Keep at 4 °C.

S-basal complete	
amount	reagent
5 mL	1 M potassium citrate solution
5 mL	trace metals solution
1.5 mL	1 M $MgSO_4$
1.5 mL	1 M $CaCl_2$
0.5 mL	5 mg/mL cholesterol in EtOH
5 mL	Penicillin-streptomycin
2.5 mL	Amphotericin B

Trace Metal Solution

To make 1 L trace metal solution:

- Add the reagents
- Fill to 1L with MilliQ water.
- Autoclave and store at 4 °C.

Trace Metals Solution	
amount	reagent
1.86 g	Disodium EDTA
0.69 g	$FeSO_4 \cdot 7H_2O$
0.20 g	$MnCl_2 \cdot 4H_2O$
0.29 g	$ZnSO_4 \cdot 7H_2O$
0.025 g	$CuSO_4 \cdot 5H_2O$

Modified C. elegans Habitation and Reproduction Medium (mCeHR)

To make 1 L mCeHR base:

- Add the choline, vitamin and growth factor mix, i-inositol, hemin chloride and 250 mL sterile water to a 1 L sterile filter bottle in a sterile biological hood.
- Apply suction to filter
- Add nucleic acid mix, mineral mix, lactalbumin hydrosylate, 50 X MEM Amino Acids Solution (Gibco), 100 X MEM Non-Essential AA Solution (Gibco), KH_2PO_4, d-Glucose and HEPES sodium salt with 256 mL autoclaved water; filter.
- Add cholesterol in ethanol without filtration.

Final volume ratio of mCeHR is 80% base to 20% non-fat milk.

mCeHR medium base	
amount	reagent
10 mL	2 mM choline
10 mL	vitamin and growth factor mix
10 mL	2.4 mM i-inositol
10 mL	1 mM hemin chloride
250 mL	sterile water
20 mL	nucleic acid mix
100 mL	mineral mix
20 mL	170 mg/mL lactalbumin hydrosylate
20 mL	50X MEM amino acids solution
10 mL	100X MEM non-essential amino acids solution
20 mL	450 mM KH_2PO_4
50 mL	1.46 M d-glucose
10 mL	1 M HEPES sodium salt
256 mL	sterile water
1 mL	5 mg/mL cholesterol

TABLES

Supplementary Table S1. Experimental details for testing potential sources (causes) of nematode counting variability.

#	Source of Counting Variability	Effect on Counting Variability	Test Procedure
1	Pipette tip size	Size exclusion bias	Query: Do differences in the sizes of the pipette tips used for transferring nematodes between containers result in a bias in the nematode counts (e.g. small pore pipette tips might potentially exclude large nematodes/adults from being counted)?<br>Test: Step 1: From the primary container (culture flask), sample aliquots were transferred (using a standard 10 µL primed pipette tip) onto glass microscope slides and the culture was counted using the counting protocol. Step 2: From the primary container (culture flask), a 1000 µL primed pipette tip was used to transfer a 1000 µL aliquot of nematode culture into a secondary container (culture flask) that was then diluted 150X with Millipore water. The primed 1000 µL pipette tip was used to transfer 1000 µL of culture into 6 wells of a 24-well microtitre plate and the plate was heated at 80 °C to heat-kill the nematodes. Each well was independently imaged using a CoolSNAPHQ2 CCD camera (Photometrics, Tucson, AZ) coupled to an automated Zeiss microscope (Axio Vert.A1, Carl Zeiss Microscopy, Oberkochen, Germany) with Zen software (Carl Zeiss Microscopy, 2012 Blue Edition). The microscope was calibrated using a stage micrometer (Electron Microscopy Services) at 5X. Images were exported as .tiff files and viewed in ImageJ. Nematode counts obtained using the counting protocol were compared to nematode counts obtained using the 1000 µL pipette tip.
2	Absence or presence of culture shaking and type of shaking	Settling bias	Query: Does settling of the nematodes in the culture flask result in a bias in the nematode counts?<br>Test: A nematode culture was gently removed from a 20 °C incubator and placed on a laboratory bench top for 5 min to settle. The culture was sampled and counted with no shaking and with minimal movement of the flask. The flask was then re-counted using Shake Style A (vigorous swirling of the flask three times clockwise and then three times counter clockwise, with the flask oriented upright and the “clock” positioned on the floor). The nematode culture was allowed to settle for 5 min, and was re-counted with Shake Style B (gentle rocking of the flask back and forth, three times in each direction, with the flask oriented upright and the rocking occurring from left to right). Flasks were gently rocked between sampling aliquots for both shaking styles.

#	Source of Counting Variability	Effect on Counting Variability	Test Procedure
3	Transfer of sample aliquots from primary to secondary to tertiary containers; nematodes sticking to pipettes	Sticking/loss during transfer	Query: Does transferring sample aliquots using unprimed, new or primed ("old") pipette tips or transferring from a primary container to a secondary or tertiary container induce a bias in the nematode counts?<br>Test 1: From the primary nematode container (culture flask), a 300 µL sample aliquot was transferred into a 1.7 mL microcentrifuge tube (secondary container). A 200 µL sample aliquot was then removed from the secondary container and transferred into a second microcentrifuge tube (tertiary container). Nematode counts obtained in secondary and tertiary containers were compared to nematode counts obtained in the primary container.<br>Test 2: From the primary container (culture flask), sample aliquots were transferred onto glass microscope slides with (1) new, unprimed pipette tips, (2) new, primed pipette tips and with (3) old (re-used), primed pipette tips. New, unprimed pipette tips were used straight out of the box. New, primed pipette tips were primed by pipetting up and down in growth culture four times.
4	Counting nematodes in different locations within a single container	Sampling location bias	Query: Does a specific sampling location within a container result in a bias in the nematode counts?<br>Test: From the primary container (culture flask), sample aliquots from the middle of the flask were transferred onto glass microscope slides and the nematodes were counted using the counting protocol. Then, a 1000 µL sample aliquot was transferred to a 1.7 mL microcentrifuge tube (tube was mixed by vortex). Sample aliquots were taken from the top, middle and bottom portions of the total sample volume in the microcentrifuge tube. Nematode counts obtained in the flask were compared to the nematode counts obtained in the 3 different locations within the microcentrifuge tube.
5	Inherent counting differences between people	Person-to-person bias	Query: Is there a significant difference between individual technicians using the same counting protocol that results in a bias in the nematode counts?<br>Test: From a single primary container (flask), three different individuals determined the nematode counts in the flask using the counting protocol; the flask contained a nematode culture prepared by a non-counting operator who hand-selected and transferred a specified number of nematodes into a known volume of culture medium. In addition, four different individuals counted a nematode culture with an unknown nematode concentration.

#	Source of Counting Variability	Effect on Counting Variability	Test Procedure
6	Transfer of different sample aliquot volumes or numbers of aliquots	Transfer bias	Query: Does transferring different sample aliquot volumes from primary container to secondary container or does transferring different numbers of aliquots induce a bias in the nematode counts?<br>Test: From the primary nematode container (culture flask), sample aliquots composed of 1 x 300 µL, 3 x 100 µL (into one tube) and 3 x 50 µL (into one tube) were transferred into separate secondary containers (1.7 mL microcentrifuge tubes) and the nematodes in each secondary container were independently counted. Nematode counts obtained in secondary containers were compared to nematode counts obtained in the primary container after correcting for dilution.
7	Order of sample aliquots (dots) on microscope slide	Location bias	Query: Does the placement order or location of the 2 µL sample aliquot (dot) on the microscope slide induce a bias in the nematode counts?<br>Test: Nematode cultures were sampled from flasks and counted with alternating dot placement (Figure 1). First, the culture was counted with the counting protocol (dot placement from spot 1 to spot 10). Next, the culture was re-counted with dots placed on slides from spot 6 to spot 10 and from spot 1 to spot 5. Three slides were prepared for each ordering sequence. The set of nematode counts from all 42 slides evaluated during the variability source examination phase of the study (dot placement from spot 1 to spot 10) were included in the final statistical analysis; these 42 slides spanned various settings of other controlled factors.

Supplementary Tables S2 through S7 and Table S9 present uncertainty intervals for the effect of various aspects of the experimental protocol. The provided intervals are intended to depict plausible values for the ratio of median nematode counts obtained between two different counting protocols. Uncertainty characterizations are dependent upon modeling choices made during analysis. Here we present uncertainty intervals obtained from two different approaches described as protocol models 1 and 2 in the Statistical Analyses portion of the Materials and Methods section of the main text.

Supplementary Table S2. Effects of Different Nematode Culture Shaking Styles on Nematode Counts. Pairs of values that are < 1 (e.g., for no shaking versus a shaking style) indicate significantly decreased counts. Pairs of values that span 1 indicate no significant difference in counts.

Shake Style Comparison	95% Posterior Credible Intervals<br>Model 1 ; Model 2
no shaking / style B	(0.616, 0.798) ; (0.640, 0.845)
style A / style B	(0.857, 1.088) ; (0.863, 1.073)
no shaking / style A	(0.638, 0.828) ; (0.663, 0.879)

Supplementary Table S3. Effects of Transferring to Secondary and Tertiary Vessels on Nematode Counts

Secondary and Tertiary Transfer	95% Posterior Credible Intervals<br>Model 1 ; Model 2
Tertiary vessel / Secondary vessel	(0.850, 1.299) ; (0.870, 1.322)
Secondary vessel / no transfer flask	(0.905, 1.245) ; (0.904, 1.223)
Tertiary vessel / no transfer flask	(0.904, 1.373) ; (0.911, 1.394)

Supplementary Table S4. Effects of Priming or Not Priming Pipette Tips

Priming Tips	95% Posterior Credible Intervals<br>Model 1 ; Model 2
New tip, no prime / New tip w\ prime	(0.508, 0.787) ; (0.519, 0.773)
New tip, no prime / Old tip w\ prime	(0.457, 0.675) ; (0.476, 0.684)
New tip w\ prime / Old tip w\ prime	(0.731, 1.056) ; (0.762, 1.065)

Supplementary Table S5. Bias from Sampling Location in Microcentrifuge Tube

Sampling Location Bias	95% Posterior Credible Intervals Model 1 ; Model 2
Bottom tube / Middle tube	(0.855, 1.290) ; (0.849, 1.347)
Bottom tube / Middle flask	(0.905, 1.368) ; (0.895, 1.402)
Bottom tube / Top tube	(1.008, 1.574) ; (0.997, 1.630)
Middle tube / Middle flask	(0.905, 1.245) ; (0.904, 1.223)
Middle tube / Top tube	(0.971, 1.492) ; (0.960, 1.476)
Middle flask / Top tube	(0.914, 1.401) ; (0.921, 1.400)

Supplementary Table S6. Effects of Transferring Nematode Culture with Different Transfer Protocols

Number of Transfers	95% Posterior Credible Intervals Model 1 ; Model 2
3 x 100 µL tube / 1 x 300 µL tube:	(0.795, 1.206) ; (0.810, 1.210)
3 x 100 µL tube / Flask	(0.843, 1.279) ; (0.858, 1.258)
1 x 300 µL tube / Flask	(0.905, 1.245) ; (0.904, 1.223)

Supplementary Table S7. Effects of Transferring Different Volumes of Nematode Culture

Volume of Transfer	95% Posterior Credible Intervals Model 1 ; Model 2
3 x 50 µL tube / 3 x 100 µL tube	(0.838, 1.308) ; (0.845, 1.291)
3 x 50 µL tube / No transfer flask	(0.883, 1.336) ; (0.883, 1.323)
3 x 100 µL tube / No transfer flask	(0.843, 1.279) ; (0.858, 1.258)

Supplementary Table S8. Bias Caused by Dot Location on Microscope Slide

Dot Position	Average Count	# Below Average of Other Dots[a]	# of Ties	# Above Average of Other Dots	Estimated Proportion (Ignoring Ties)*	95% CI Lower	95% CI Upper	P-value
1	17.1	43	1	19	0.306	0.196	0.437	0.003
2	19.8	33	0	30	0.476	0.349	0.606	0.801
3	19.2	34	0	29	0.46	0.334	0.591	0.615
4	19.7	30	0	33	0.524	0.394	0.651	0.801
5	19.7	29	1	33	0.532	0.401	0.66	0.704
6	19.7	26	0	37	0.587	0.456	0.71	0.207
7	19.5	29	0	34	0.54	0.409	0.666	0.615
8	18.5	32	0	31	0.492	0.364	0.621	1
9	18.7	34	2	27	0.443	0.315	0.576	0.443
10	16.3	42	1	20	0.323	0.209	0.453	0.007

*The proportion listed in row i corresponds to fraction of slides for which the count observed at dot position i was greater than the average count across the other nine positions on the same slide. If dot location had no effect on nematode count, one would expect this value to vary randomly around 0.5. Confidence intervals from dot positions 1 and 10 fall entirely below 0.5, which indicates a potential negative bias.
a/ Out of a total of 63 measurements.

Supplementary Table S9. Bias between Operators

Operator	95% Posterior Credible Intervals Model 1 ; Model 2
1 / 2	(0.957, 1.197) ; (0.954, 1.196)
1 / 3	(0.943, 1.178) ; (0.940, 1.179)
1 / 4	(0.853, 1.170) ; (0.837, 1.184)
2 / 3	(0.885, 1.094) ; (0.882, 1.101)
2 / 4	(0.787, 1.094) ; (0.774, 1.105)
3 / 4	(0.801, 1.112) ; (0.791, 1.122)

Supplementary Table S10. Days to Hatching (time from embryo to juvenile nematode)

Number of Days to Egg Hatching	Number of Observations in mCeHR cultures	Number of Observations in S-Basal cultures
4 d	1	5
5 d	4	0

FIGURES

Supplementary Figure 1. Schematic of for Sampling Nematodes from Liquid Culture. The sampling protocol for counting nematodes in liquid culture is briefly described. The three most important aspects of sampling are italicized in the text and emphasized in red in the figure. These include swirling the sample, priming the pipette tip in the culture medium and removing aliquots from the middle of the culture (as opposed to the top). We also recommend only submersing the tip about halfway into the medium, and not allowing the pipette shaft to come into contact with the medium to avoid contamination.

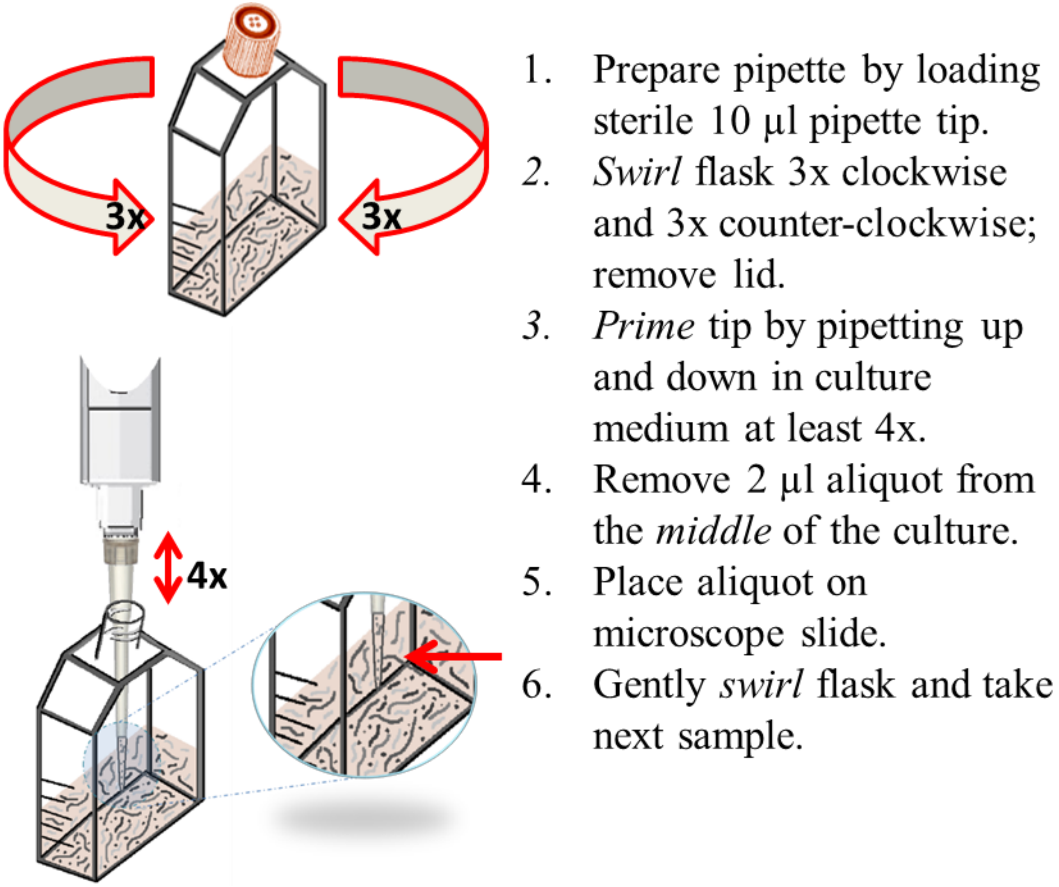

Figure S1. Schematic for sampling nematodes from liquid culture. The sampling protocol for counting nematodes in liquid culture is briefly described. The three most important aspects of sampling are italicized in the text and emphasized in red in the figure. These include swirling the sample, priming the pipette tips in the culture medium and removing aliquots from the middle of the culture (as opposed to the top).

COUNTING PROTOCOL FOR NEMATODES IN LIQUID CULTURE

The protocol described is for 10 mL cultures grown in 25 cm^2 tissue culture flasks. Modified CeHR cultures should only be opened in a sterile BSL-2 hood to ensure sterility (airflow typically turned on 10 min before use).

1. Spray the hood with 70 % volume fraction ethanol and wipe accessible surfaces with a paper towel. S-basal complete cultures can be opened and passaged on a bench-top sprayed with 70 % volume fraction ethanol.
2. Place a sterile 10 µL pipette tip onto a 10 µL pipette. Shake the flask gently, e.g. by swirling medium around inside the flask three times clockwise and then three times counter clockwise.
3. Unscrew and remove flask cap and prime pipette tip by pipetting the contents up and down in the nematode culture at least four times. Tips can be reused for all 10 dots in one counting replicate.
4. Remove a 2 µL aliquot from the culture, check to make sure the sample is in the pipette tip, and transfer the sample onto a sterile, glass microscope slide at the top left corner of the slide. After removing the aliquot, gently shake the flask (3x3, as described above) before sampling again. Place dots onto the glass slide in the following order: dot 1 is placed at the top left of the slide near the slide label or edge. Dots 2 to 5 are placed to the right of each preceding dot. Dot 6 is placed on the bottom right of the slide (below dot 5). Dots 7 to 10 are placed to the left of each preceding dot.
5. When 10 x 2 µL dots are on the slide, examine the slide using a light microscope and count nematodes at 40X magnification (4X objective and 10X eyepiece). Beginning with the first dot, count the number of nematodes of all sizes, including juveniles and adults. It is also possible to record dead nematodes, quantify nematodes of different stages and identify "bag of nematodes", or nematode adults with internal hatched neonates (indicative of a problem with the organism and potentially with the culture).
6. If the nematode culture is too dense to accurately count, dilute the culture as described below.
7. Record counts and dilutions.
8. Wipe off the microscope slide and spray with with 70% volume fraction ethanol when counting is completed. Slide can be reused.

To dilute cultures:

1. Swirl culture flask three times clockwise and three times counter clockwise with the flask oriented upright; remove the flask cap.
2. Prime an appropriate sized pipette tip (e.g. 200 µL or 1000 µL) at least four times with the culture medium.
3. Transfer 50 µL or 300 µL of the culture medium into a 1.7 mL microcentrifuge tube.
4. Add an appropriate volume of sterile water into the tube and record the volume and dilution factor.
5. Cap the microcentrifuge tube and vortex mix the sample for 4 s at a low to medium speed.

Dilutions are recommended for nematode cultures that are too dense to count easily (cultures that contain ≥ 15 to 20 nematodes per μL or ≥ 30 to 40 nematodes per dot). It is strongly recommended that cultures are kept at < 20 nematodes per μL in order to ensure optimal health of the culture.

REFERENCES

1 Samuel, T. K., Sinclair, J. W., Pinter, K. L. & Hamza, I. Culturing *Caenorhabditis elegans* in axenic liquid media and creation of transgenic worms by microparticle bombardment. *J. Vis. Exp.*, e51796 (2014).
